# Assisted reproductive techniques in Latin America: The Latin American registry, 2016

**DOI:** 10.5935/1518-0557.20190037

**Published:** 2019

**Authors:** Fernando Zegers-Hochschild, Juan Enrique Schwarze, Javier A Crosby, Carolina Musri, Maria Teresa Urbina

**Affiliations:** 1 Unit of Reproductive Medicine, Clínica Las Condes, Santiago, Chile; 2 Program of Ethics and Public Policies in Human Reproduction, University Diego Portales, Santiago, Chile; 3 Latin American Network of Assisted Reproduction (REDLARA), Montevideo, Uruguay; 4 Unifertes, Caracas, Venezuela

**Keywords:** ART, assisted reproductive technologies, multiple pregnancy, outcome, registry

## Abstract

**Research question:**

What was the utilization, effectiveness and perinatal outcome of assisted reproductive technologies (ART) performed in Latin America during 2016

**Design:**

Retrospective collection of multinational data on ART performed in 178 institutions from 15 Latin American countries.

**Results:**

We are reporting 85,474 initiated cycles, 15,070 deliveries and 18,182 babies born in this period. Of all fresh autologous IVF/ICSI cycles, 40.9% were performed in women aged 35–39 years, and 31.1% in women aged ≥40 years. After removing freeze-all cycles, delivery rate per oocyte retrieval was 20.31% for ICSI and 21.85% for IVF. Fresh single embryo transfer including all age categories represented 22.96%, with a 15.35% delivery rate per transfer. Double embryo transfer represented 61.58% of transfers, with a 27.62% delivery rate per transfer. Multiple births included 18.12% twins and 0.55% triplets and higher. In oocyte donation, delivery rate per transfer was 32.89%, with a twin and triplet rate of 23.48% and 0.73%, respectively. Overall, preterm deliveries reached 17.11% in singletons, 65.69% in twins and 95.51% in triplets. Perinatal mortality was 8.0 ‰ in singletons, 19.0 ‰ in twins, and 62.3 ‰ in high-order multiples.

**Conclusions:**

The number of initiated cycles continues to increase. Compared with previous years, the number of embryos transferred decreased while the proportion of single embryo transfers increased with a drop in multiple births. It is mandatory to stimulate health care providers and consumers to continue in this trend.

## INTRODUCTION

This is the 28^th^ report of the Latin American Registry of Assisted Reproduction (RLA) established in 1990 as the first multinational and regional registry of assisted reproductive techniques (ART). Previous reports, from 1990 to 1998, are available as printed copies; from 1999 to 2009 as PDF files, which can be downloaded (www.redlara.com). Since 2012, reports are published simultaneously in *Reproductive BioMedicine Online* and in *JBRA Assisted Reproduction*, the official journal of the Latin American Network of Assisted Reproduction (REDLARA). This report presents information on utilization/availability, effectiveness, safety and perinatal outcomes of ART treatment initiated between 1 January and 31 December 2016, and babies born up to September 2017.

## MATERIALS AND METHODS

 Data on ART were collected from 178 centers in 15 countries in Latin America (Supplementary Table 1), covering fresh autologous cycles of IVF and intracytoplasmic sperm injection (ICSI); frozen autologous embryo transfer (FET); oocyte donation (OD) including the transfer of both fresh and frozen/thawed embryos; fertility preservation (FP); and vitrified/warmed oocyte cycles (OTHER), both autologous and heterologous.

This report includes treatments started between 1 January 2016 and 31 December 2016. Data on pregnancy and neonatal outcomes are obtained from follow-up of cohorts treated during this period.

As part of the accreditation program, all participating institutions agree to have their data registered and published by the RLA. Therefore, no other consent form was requested for the scientific disclosure of these data.

The method of collecting data in 2016 resembles previous years (Zegers-Hochschild *et al*., 2018; 2019), making results comparable. Definitions used refer to the latest publication of the International glossary on Infertility and Fertility Care (Zegers-Hochschild *et al*., 2017). When calculating clinical pregnancy or delivery rates per oocyte pick-up, cases of total embryo freezing were not included in the calculation.

Cumulative live birth rate was calculated, as described by Maheshwari *et al*. (2015) from cycles taken place between 2012 and 2016. We considered the first delivery after transfer of either fresh or frozen/thawed embryos obtained after a reference oocyte pick up. Each patient was identified by a personal identification number and date of birth. The identification number is not yet universal in Latin America, so not all patients could be followed and it is also possible that cross border reproductive treatments could partially influence results, but the numbers should be small. Furthermore, it was not possible to follow up individual patients in all reporting institutions; only those in which a consistent ID number was used throughout the study period (2012 and 2016).

In order to test for the effect of age, number of embryos transferred and state of embryo development at transfer on the delivery rate per embryo transfer, logistic regression analysis was performed in both fresh and OD cycles. When appropriate, a chi-squared test was used to analyze independence of categorical variables. A *p*-value less than 0.05 was considered statistically significant.

## RESULTS

### Participation

178 centers in 15 countries reported ART procedures performed during 2016. This represents approximately 70% of centers in the region. The majority of centers were located in Brazil (n = 62), followed by Mexico (n = 33) and Argentina (n = 27) (Table 1). In comparison with 2015, 6 centers stopped reporting, having contributed with only 1,200 cycles in 2015, which represents 1.4% of all cycles reported in 2016. Furthermore, new centers incorporated between 2015 and 2016 contributed with more than 3,800 of the 10,353 new cycles reported in this period. In many cases, the proportion of centers reporting is not paralleled with the number of cycles covered by the registry. An example is Argentina where only 27 out of 62 centers (43.5%) reported to REDLARA; however, 20,793 out of 23,663 (87.9%) of the initiated cycles performed in Argentina are in fact covered by the Latin American registry.

**Table 1:** Assisted reproduction technique procedures reported to RLA in 2016

Country	Centres	FP	Fresh	FET	OD	Other	Total
Argentina	27	851	11,192	4,535	3,880	335	20,793
Bolivia	3	1	412	63	180	0	656
Brazil	62	1,919	20,027	9,818	2,526	975	35,265
Chile	10	385	2,253	1,156	862	217	4,873
Colombia	12	49	1,254	463	540	71	2,377
Ecuador	6	116	537	269	250	74	1,246
Guatemala	1	3	106	45	43	0	197
Mexico	33	247	5,984	2,535	3,389	327	12,482
Nicaragua	1	0	109	7	14	0	130
Panama	3	25	480	156	149	15	825
Paraguay	1	14	109	69	32	4	228
Peru	10	714	1,519	591	844	516	4,184
Rep. Dominicana	1	0	39	18	43	0	100
Uruguay	1	17	601	218	133	10	979
Venezuela	7	24	633	180	300	2	1,139
Total	178	4,365	45,255	20,123	13,185	2,546	85,474

FP, fertility preservation; Fresh, initiated IVF/ICSI cycles; FET, frozen autologous embryo transfer; OD, transfer of fresh or frozen embryos due to oocyte donation; Other includes frozen thawed oocytes own and donated.

### Size of participating institutions 

A total of 85,474 initiated cycles were reported (13.8% more than the previous year), corresponding to the sum of fresh autologous IVF/ ICSI, FET, OD, FP and embryo transfer cycles of embryos resulting from vitrified-warmed oocytes, either autologous or donated, grouped as OTHER.

The mean number of initiated cycles by institution was 480.2, with wide variation; 15.6% performed ≤100 cycles; 31.8% between 101 and 250 cycles; 22.9% between 251 and 500 cycles; 18.4% between 501 and 1000 cycles, and 11.3% >1000 cycles. Overall, the major contributor was Brazil with 41.3%, followed by Argentina with 24.3% and Mexico with 14.6% of initiated cycles.

### Number of treatment cycles per technique and access to treatment

Out of 85,474 initiated cycles, 45,255 corresponded to fresh autologous IVF/ICSI (52.9%); 20,123 corresponded to FET (23.5%); 13,183 to OD (15.4%), 4,363 to FP (5.1%), and 2,546 cycles reported as OTHER which include the transfer of embryos resulting from vitrified/warmed oocytes (2.9%).

Of the 45,255 initiated fresh autologous IVF/ICSI cycles, at least one mature oocyte was recovered in 42,146 aspirations (93.1% of cases). The preferred method for insemination was ICSI (86.6%). Overall, at least one embryo was transferred in 24,451 cases. The main reasons for no embryo transfer were: 12,730 cases of total embryo freezing, 2,265 cases of abnormal in-vitro embryo development, and 1,327 cases of total fertilization failure corresponding to 3.1% of inseminations. In addition, there were 1069 cases of no oocytes retrieved, 622 cases of no mature oocytes retrieved, 857 cases where only abnormal embryos were obtained after PGT, 470 cases where the reason for no embryo transfer included abnormal oocyte after PGT and other conditions of unknown origin.

Utilization of ART is still very low in Latin America. In 2016, reached 136 initiated cycles per million people living in the 15 countries reporting to RLA, with great variations between countries. Reporting ranged from 12 and 21 cycles per million in Guatemala and Nicaragua respectively, to 474 and 284 cycles per million in Argentina and Uruguay. It is important to mention that not all centers performing ART report to the RLA. It is estimated that overall, 70% of centers report, including the majority of institutions performing ≥1000 cycles per year. Therefore, the coverage in number of initiated cycles is estimated to be in the order of 80% globally. Argentina is the country with highest utilization and the first in Latin America, followed by Uruguay, to have a law providing universal care to infertility treatments

### Age distribution

The mean age of women undergoing fresh autologous IVF/ICSI was 36.9 years (SD 4.5). The highest proportion of cycles was performed in women aged 35 to 39 years (40.9%), followed by (31.1%) of women aged ≥40 years. Therefore, 72.0% of women using autologous ART were ≥35 years. The mean age of women undergoing OD was 41.6 (SD 5.0); and the majority of cycles (56.5%) were performed in women aged ≥42 years

### Outcome of pregnancies and deliveries 

In the present year, 23,894 clinical pregnancies were reported, of which 1,725 (7%) were lost to follow-up. Thus, the analysis of outcome variables should not be affected by these losses. Table 2 shows the clinical pregnancy rate (CPR) and delivery rate (DR) per oocyte pick- up (OPU) in fresh autologous IVF/ICSI cycles. Considering that the number of procedures are much higher in ICSI than IVF, results in terms of CPR per OPU were not significantly higher in IVF than in ICSI cycles (28.31% and 27.39%) but the DR per OPU was higher in IVF compared with ICSI 21.85% and 20.31% respectively, *p*<0.0285. When calculated by transfer, the DR per ET in IVF and ICSI were almost identical, 24.6% and 24.9% respectively.

**Table 2:** Clinical pregnancy rate and delivery rate in IVF and intracytoplasmic sperm injection cycles in 2016

Assisted reproduction technique procedure	Oocyte retrievalª	Clinical pregnancy rate per oocyte retrieval (%)	Delivery rate per oocyte retrieval (%)
ICSI	25480	6979 (27.39)	5174 (20.31)
IVF	3931	1113 (28.31)	859 (21.85)
*p*-value	N.A.	0.2427	0.0285

ª Oocyte retrieval with at least one mature oocyte

**Figure 1:**
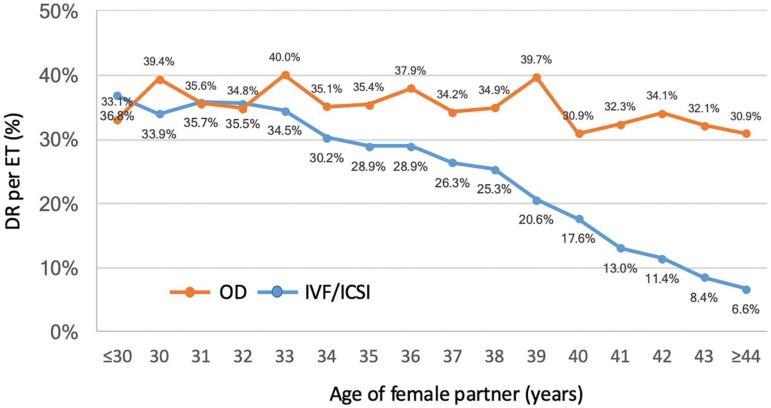
Delivery rate per embryo transfer according to woman´s age in IVF/ICSI and OD cycles, RLA 2016

As expected, both CPR and DR per ET were much higher after the transfer of donated oocytes (OD) than in autologous reproduction, reaching 44.96% and 32.89%, respectively (Table 3). Thus, outcome after OD is only marginally affected by the age of the recipient. (Figure 1).

**Table 3:** Clinical pregnancy rate and delivery rate by embryo transfer in oocyte donation and FET cycles in 2016

Assisted reproduction technique procedure	Embryo transfer	Clinical pregnancy per embryo transfer (%)	Delivery rate per embryo transfer (%)
Oocyte donation	10476	4710 (44.96)	3446 (32.89)
Frozen-thawed embryo transfer	19608	6982 (35.61)	4993 (25.46)

**Figure 2:**
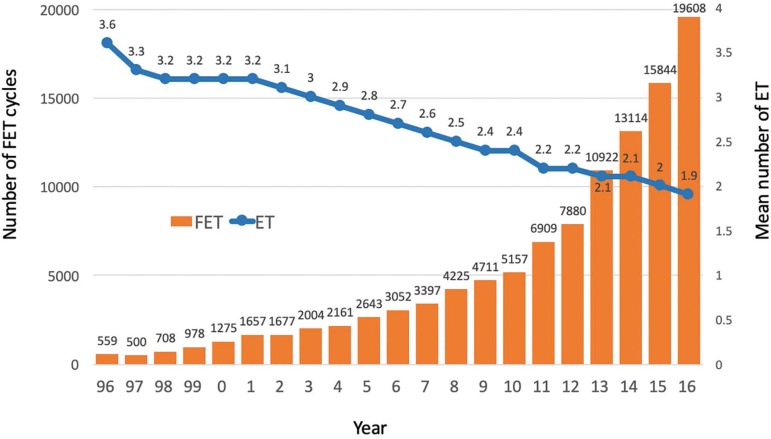
Number of FET cycles and mean number of embryo transferred according to year. RLA, 1996 - 2016

The number and proportion of FET cycles have increased yearly since 1996; with an increment of 22.6% between 2015 and 2016, accompanied by a proportional drop in the mean number of embryos transferred reaching 1.9 in 2016. (Figure 2).

 In FET cycles, the overall CPR and DR per transfer was 35.61% and 25.46%, respectively (Table 3). As seen in Tables 3 and 4, the CPR in FET is significantly higher than in fresh transfers (35.6% and 33.1% respectively, *p*<0.0001); nonetheless, the DR per transfer did not differ in FET and fresh transfers (25.5% and 24.7% respectively).

**Table 4:** Clinical pregnancy rate, delivery rate and gestational order according to the number of embryos transferred in IVF and intracytoplasmic sperm injection cycles in 2016

Number of transferred embryos	Total embryo transfer		Clinical pregnancy		Deliveries							
	Number	%	Number	%	Number of deliveries	Delivery rater per embryo transfer (%)	Singleton (n)	Singleton (%)	Twin (n)	Twin (%)	≥Triplets (n)	≥Triplets (%)
1	5,614	22.96	1,215	21.64	862	15.35	847	98.26	15	1.74	0	0.00
2	15,057	61.58	5,557	36.91	4,158	27.62	3,274	78.74	867	20,85	17	0.41
3	3,442	14.08	1,217	35.36	945	27.45	732	77.46	199	21.06	14	1.48
≥4	338	1.38	100	29.59	68	20.12	54	79.41	12	17.65	2	2.94
Total	24451	100	8089	33.08	6033	24.67	4907	81.34	1093	18.12	33	0.55

### Number of embryos transferred, deliveries and multiple births after fresh autologous IVF/ICSI according to the age of women

In women ≤34 years, there were 7,082 fresh transfers. The mean number of embryos transferred was 1.91 (range 1 to 5). In this age group, 19.7% were single embryo transfers (SET) and 8.2%, elective (eSET). Double embryo transfers (DET) corresponded to 69.9% of transfers and elective (eDET) was 33.5%. The transfer of three embryos (TET) and 4 or more, was performed in 10% and 0.4% of cases.

In women aged 35 to 39 years, the mean number of embryos transferred was 1.95 (range 1 to 5). In this age group, 22.4% were SET and 5.2% eSET. DET corresponded to 61.4% of transfers and eDET was 23.3%. The transfer of three embryos (TET) and 4 or more, were performed in 15.5% and 0.7% of cases.

In women ≥40 years of age, the mean number of embryos transferred was 1.95 (range 1 to 5). In this age group 27.7% were SET and 2.2% eSET, 52.8% DET and 11.9% eDET, and 16.1% TET; while the transfer of four or more embryos occurred in 3.4% of transfers.

**Figure 3:**
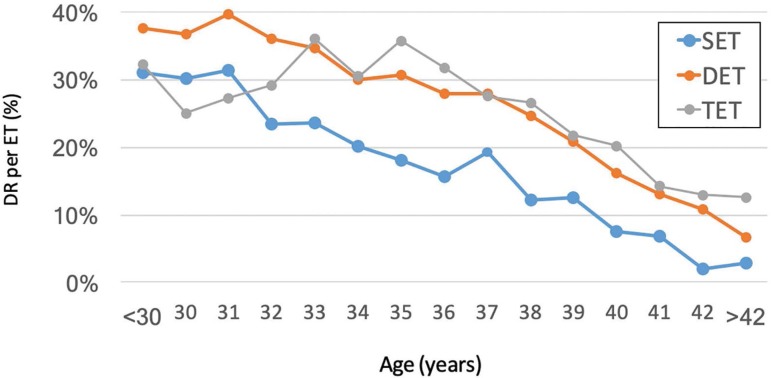
Delivery rate per embryo transfer after the transfer of one (SET), two (DET) and three embryos (TET), according the woman´s age, RLA IVF/ICSI 2016

Figure 3 shows the delivery rate according to the age of female partner, after the transfer of 1, 2 and 3 embryos. As seen, irrespective of the age of the female partner, DR is significantly higher after the transfer of 2 over 1 embryo (OR 2.038 95% CI 1.865-2.227). However, the transfer of 3 embryos does not increase DR over the transfer of 2 embryos (OR 0.929 95% CI 0.842-1.025).

Table 4 summarizes the overall number of embryos transferred and multiple births after fresh autologous IVF/ICSI. The mean number of embryos transferred was 1.94 (range 1 to 5). There were 5,614 SET (22.96%), of which only 1,276 were eSET (5.22%). There were 15,057 DET (61.58%), of which 5,669 (23.02%) were eDET.

Overall, the CPR and DR per ET reached 33.08% and 24.67%, respectively. In cases of eSET, the DR per ET reached 29.39%, increasing to 35.95% in eDET. In terms of multiple births, of the 6,033 fresh autologous IVF/ICSI deliveries registered, 81.34% were singletons, 18.12% were twins, and 0.55% were triplets or more.

### Number of embryos transferred, deliveries and multiple births after OD and FET 

Table 5 summarizes the number of embryo transfers and multiple births in OD (fresh and FET), where the mean number of embryos transferred reached 1.93 (range 1 to 5). There were 2,305 SET, which correspond to 22.0% of ET and 673 were eSET, representing 6.42% of all ET/OD. There were 6,648 DET, which correspond to 63.45% of ET, and 4,619 were eDET, representing 44.09% of all transfers in OD.

**Table 5:** Clinical pregnancy rate, delivery rate and gestational order according to the number of embryos transferred in fresh and frozen oocyte donation cycles in 2016

Number of transferred embryos	Total embryo transfer		Clinical pregnancy		Deliveries							
	Number	%	Number	%	Number of deliveries	Delivery rater per embryo transfer (%)	Singleton (n)	Singleton (%)	Twin (n)	Twin (%)	≥Triplets (n)	≥Triplets (%)
1	2305	22.00	927	40.22	622	26.98	610	98.07	12	1.93	0	0.00
2	6648	63.46	3038	45.70	2225	33.47	1622	72.90	600	26.97	3	0.13
3	1471	14.04	723	49.15	581	39.50	364	62.65	195	33.56	22	3.79
≥4	52	0.50	22	42.31	18	34.62	16	88.89	2	11.11	0	0.00
Total	10476	100	4710	44.96	3446	32.89	2612	75.80	809	23.48	25	0.73

Overall, the CPR and DR per ET were 44.95% and 32.89%, respectively. Of the 3,446 deliveries registered, 75.79% were singletons, 23.48% were twins and 0.73% were triplets and higher. Furthermore, DR/ET was slightly affected by the age of the oocyte recipient (OR 0.98 95% CI 0.97-0.98) (Figure 1).

In FET cycles, Table 6 summarizes the number of embryos transferred, where the mean number of embryos transferred reached 1.79 (range 1 to 4). There were 6,082 SET, which correspond to 31.01% of ET. There were 11,628 DET, which correspond to 59.30% of ET. Overall, the CPR and DR per ET reached 35.61% and 25.46%, respectively. Of the 4,993 deliveries registered, 81.68% were singletons, 17.68% were twins, and 0.64% were triplets and higher.

**Table 6:** Clinical pregnancy rate, delivery rate and gestational order according to the number of embryos transferred in frozen embryo transfer cycles in 2016

Number of transferred embryos	Total embryo transfer		Clinical pregnancy		Deliveries							
	Number	%	Number	%	Number of deliveries	Delivery rater per embryo transfer (%)	Singleton (n)	Singleton (%)	Twin (n)	Twin (%)	≥Triplets (n)	≥Triplets (%)
1	6,082	31.02	1,884	30.98	1,276	20.98	1,239	97.10	37	2.90	0	0.00
2	11628	59.30	4462	38.37	3246	27.,92	2487	76.62	742	22.86	17	0.52
3	1793	9.14	605	33.74	448	24.99	333	74.33	100	22.32	15	3.35
≥4	105	0.54	31	29.52	23	21.90	19	82.61	4	17.39	0	0.00
Total	19608	100	6982	35.61	4993	25.46	4078	81.67	883	17.68	32	0.,64

### Influence of stage of embryo development at transfer 

Overall, 49.64% of ET were performed at the blastocyst stage. The proportion of blastocysts transfers in FET (67.64%) was double the proportion in fresh autologous IVF/ICSI (30.64%). In OD cycles (both fresh and frozen/thawed embryo transfers), the proportion of blastocyst transfers reached 53.35%.

Blastocyst transfers were always associated with an increase in the DR/ET compared with cleavage-stage embryos, irrespective of whether fresh or frozen and the number of embryos transferred. In fresh autologous IVF/ICSI, the DR of 7,506 transfers of blastocysts was 31.16% compared with 21.77% after the transfer of 16,967 cleaving embryos (*p*<0.0001). In OD, the DR/ ET was 40.61% in blastocyst transfers and 27.84% in cleaving embryo transfers (*p*<0.0001). In FET, the proportion was 40.51% and 28.74% respectively, (*p*<0.0001)

### Perinatal outcome and complications 

Table 7 summarizes perinatal mortality. Data was available from 15,070 births and 18,182 babies born. The perinatal mortality increased from 8.2‰ births in 12,055 singletons, to 19.31‰ in 5,838 twins and 63.2‰ in 289 triplets and higher.

**Table 7:** Perinatal mortality according to gestational order in 2016

	Singleton	Twin	≥ Triplets
Livebirth*	11,959	5,727	271
Stillbirth	33	23	7
Early neonatal death	63	88	11
Perinatal Mortality**	8.0‰	19.0 ‰	62.3‰

(*) Early neonatal death are excluded

(**) Perinatal Mortality=(stillbirth+early neonatal death)/( livebirth + stillbirth + early neonatal death)

Gestational age at delivery was reported in 13,251 deliveries (87.9% of all deliveries). The mean gestational age at delivery was 37.7 (SD 2.2) weeks in singletons, 35.1 (SD 2.8) weeks in twins, and 32.3 (SD 3.8) weeks in triplets and higher. The overall risk of preterm birth (gestational weeks 22-36) increased from 17.11% in singletons, to 65.69% in twins, and 95.51% in triplets and higher. Furthermore, the risk of very preterm birth (gestational weeks 22-27) increased from 0.83% in singleton to 2.48% in twins and to 5.62% in triplets and higher. Table 8 shows the weight of babies born after fresh, frozen/thawed and fresh OD treatments, according to the order of gestation. As expected, the weight of singletons born after FET (3,160±547) is significantly higher than babies born after fresh transfer (3,055±550; *p*<0.00001). A similar situation occurs after twin births.

**Table 8:** Neonatal Outcome in Latin America, 2016

ART procedure	Singleton		Twin		≥Triplets	
	Weeks of gestation	Weight	Weeks of gestation	Weight	Weeks of gestation	Weight
IVF/ICSI	37.8	3055.16±550.6	35.2	2254.05±515.8	32.6	1711.20±450.2
FET	37.7	3160.10±547.7	35.1	2338.99±476.4	31.8	1565.32±460.4
OD	37.3	2975.39±581.3	35.1	2265.37±475.9	32.5	1477.00±450.1

Maternal complications are not presented due to lack of confidence in the completeness of data collected by RLA.

### Total embryo freezing 

12,730 cycles of total embryo freezing were reported, 44.6% more than in 2015. On average 4.1 embryos (SD 3.2) were cryopreserved. Overall, 5,041 cycles of FET were performed, generating 1,579 deliveries and the DR/ET of 31.3%: This is higher than a mean of 25.46% of DR/ET in FET cycles that follow fresh cycles (*p*<0.00001). A second FET attempt was reported in 994 cases from the same cohort, with 262 subsequent deliveries, the DR/ET in this attempt was 26.35%. Therefore, adding all transfers from this subset of total embryo freezing, the DR/ET adds to 30.5%.

### Preimplantation genetic testing (PGT) 

The RLA registers PGT-M and PGT-A together. 122 centers reported these procedures in 3,775 fresh cycles (8.6% of OPU); 1,124 (4.6% of transfers) using frozen-thawed embryos and 248 (2.8% of transfers) in OD. The mean age of women undergoing PGT was 38.5 (SD 4.0) among fresh cycles and 37.9 (SD 4.5) in FET.

In the case of fresh cycles, the mean number of embryos biopsied was 3.1 (SD 2.2), and the mean number of normal embryos was 1.1 (SD 1.4). In FET cycles, the mean number of embryos biopsied was 3.4 (SD 2.5), and the mean number of normal embryos was 1.8 (SD 1.3). In OD, the mean number of embryos biopsied was 4.8 (SD 2.7), and the mean number of normal embryos was 2.6 (SD 1.9). The DR/ET was 22.13% in fresh IVF/ICSI, 36.83% in FET and 34.45% in OD.

### Miscarriage

Miscarriage rate in 8,092 pregnancies resulting from autologous fresh embryo transfer and 6,982 pregnancies of FET were 17.4% and 17.9%, respectively. As expected, miscarriage rate in a total of 4,710 OD was lower both in fresh transfers (15.9%) and in frozen/thawed OD (16.1%). Furthermore, in 672 cases of OD using FTO, miscarriage rate was the lowest of all, 12.5% The miscarriage rate using PGT reached 13.4% in pregnancies after FET and 12.5% in OD.

### Fertility preservation (FP)

A total of 4,365 initiated cycles for FP were reported in 2016. The mean age of women was 36.2 (SD 5.5) years. No oocytes were available for freezing in 191 follicular aspirations (4.4%). The mean number of oocytes cryopreserved was 7.4 (SD 6.5). In cases where the indication for FP was recorded, the majority were related to the desire to postpone pregnancy, 2,660 cases representing 63.7%; while cancer-related factors were reported in 377 cases (9.0%); risk of premature ovarian insufficiency in 175 (4.2%) cases and other reasons in 962 cases (23.0%).

### Cumulative delivery rate (CDR)

**Figure 4:**
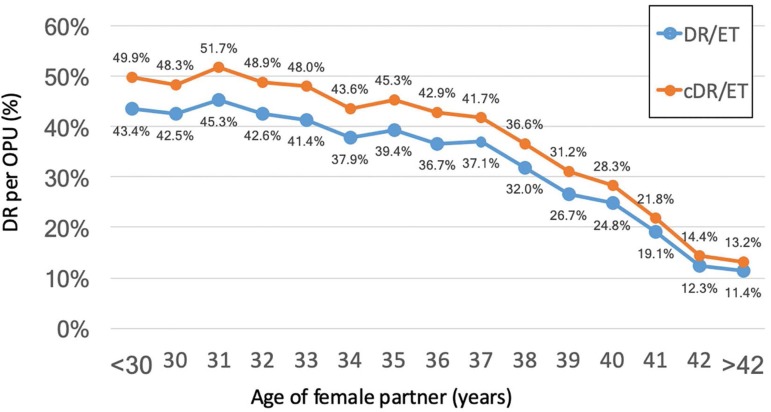
Cumulative delivery rate (cDR/ET) and delivery rate per fresh embryo transfer (DR/ET) from 2012 to 2016, according to age of the female partner.

We were able to follow up the outcome of fresh embryo transfers and their consecutive FET in 48,214 patients between 2012 and 2016. This cohort included only women having surplus frozen embryos resulting from their fresh transfer. Taking all patients together, the DR/OPU increased from 36.6% to 42.0% (RR 1.15; 95%CI 1.13-1.17; *p*<0.0001). The cumulative DR per OPU stratified by the age of female at the time of OPU is shown in Figure 4. The increment in DR when adding FET over fresh transfers was inversely correlated to the age of the female partner. The OR for delivery was 1.3 in women <35 years (95% CI 1.2 to 1.3), 1.2 in women 35 to 39 (95% CI 1.2 to 1.3) and 1.1 in women >39 (95% CI 1.1 to 1.3).

## DISCUSSION

The present report is the 28^th^ consecutive annual RLA report on ART procedures performed in Latin America. In spite of the fact that no more than 70% of centers available in the region are actually reporting to the registry, it is estimated that nearly 80% of the cycles performed in the region are included in this report. An exemplifying case is Argentina, the second largest contributor representing 25% of cycles reported to RLA. The proportion of centers that Argentina is reporting to RLA represent only 46% of existing centers in the country; however, RLA collects data from 20,793 cycles out of a total of 23,660 performed in the country. Therefore, 87.9% of cycles performed in Argentina are reported by RLA. This situation is similar in several countries including Peru, Chile among others.

Overall, the number of reported initiated cycles increased by 14% with respect to the previous year (Zegers-Hochschild *et al*., 2017). The rise in the number of initiated cycles, results in part by the contribution of new centers and also by an increase of 41.2% in the number of cycles with total embryo freezing as well as a rise of 22.6% in FET cycles, which in part is associated with a rise in the proportion of SET and DET.

In spite of this global rise in ART cycles, utilization in Latin America (136 initiated cycles/million population) remains very much under the threshold of 1,500 cycles per annum per million inhabitants proposed by the ESHRE Capri Group, in order to fulfil the needs of a population (ESHRE Capri Workshop Group, 2001).

In the vast majority of countries, ART is provided by private institutions but health insurances do not cover infertility treatments. Therefore, only a small proportion of infertile couples can afford out of pocket funding; but there are exceptions. Argentina was the first country that in 2013 legislated in favor of universal access to infertility treatment including ART. Correspondingly, it is the country with the highest utilization rate, reporting 474 cycles/million populations, and this is increasing every year. This reproductive rights initiative was then followed by Uruguay, which has the second highest utilization rate with almost 300 cycles per million populations. This relationship confirms the importance of financial affordability in the utilization of ART. In countries with strong economic inequalities, the number of couples who can afford treatment are few. Public policies providing partial or complete financial support to couples requiring ART are needed in order to increase utilization and decrease the burden generated by infertility *per se*, as well as the burden which results from lack of access in a society with profound inequalities.

The reporting of efficacy of ART can be presented in different ways. Because the number of freeze-all cycles has increased, the calculation of outcome (pregnancy or livebirth) per OPU need to exclude freeze-all cycles. The overall DR per OPU for fresh non- donor cycles in IVF and ICSI was 21.85% and 20.31%, respectively (Table 2). The delivery rate per transfer is higher in FET than fresh cycles and this difference is especially evident in SET where the DR/ET after FET was 20.98% compared with only 15.35% after fresh SET. A plausible explanation would be the higher proportion of blastocyst transfer in FET cycles, 67.4% of transfers, compared with 30.64% in fresh transfers. We have simultaneously shown that in autologous IVF as in OD, the CPR and DR is significantly higher when blastocysts are transferred compared with the transfer of cleaving embryos. This better outcome after FET is also seen in cases of total embryo freezing. As in our previous report, the CPR and DR per ET are even higher in cases of total embryo freezing than in frozen transfers after a failed fresh transfer. Of course, FET after fresh transfer can entitle a negative selection of embryos and a negative selection of the population, since those women were not pregnant in their first event; while in total embryo freezing, women were not previously exposed to pregnancy with that particular cohort of oocytes/embryos.

Preimplantation genetic testing (PGT) is also increasing in Latin America. It is now reported in 122 out of 178 institutions. When comparing outcome of pregnancies, miscarriage rate in 15,074 pregnancies after fresh and frozen/thawed autologous cycles was 17.7% compared with 12.3% in 512 pregnancies where PGT was performed. Although the numbers are still relatively low, more and more, women and men in Latin America are seeking for assurance of delivering "normal" offspring, even in cases of OD (2.8% of PGT performed in OD cycles).

Latin America has much place to improve. Starting with increasing access to treatment, which shall not only decrease the burden of disease, but also bridge the abysm between the rich and the poor who suffer from infertility.

Given the positive relationship between an increased success rates in FET cycles over fresh and in blastocyst transfers over cleaving embryos transfers, clinicians need to improve patient selection and their preparation for IVF, eliminate comorbidity whenever possible and incorporate adequate stimulation protocols in order to provide good quality gametes. On the other hand, embryologists need to generate appropriate long-term culture conditions and optimize *in vitro* embryo handling in order to allow more patients to reach blastocysts compatible with SET. Only then, we will avoid the transfer of three embryos and will keep moving towards a success rate based on cumulative live births rather than pregnancy at the first attempt. This concept is further demonstrated by the lack of improvement in birth rates after the transfer of 3 over 2 embryos (Fig. 3) and most of all, the significantly higher cumulative delivery rates when frozen transfers follow fresh transfers in women up to the age of 40, beyond which, results have less clinical significance.

## Supplementary material

**Supplementary Table 1:** Centres reporting to Latin America Registry of ART in 2016

ARGENTINA	
•	Instituto de Fertilidad Asistida
•	Centro de Estudios en Ginecología y Reproducción (CEGYR)
•	Centro de Salud Reproductiva (CER)
•	CERF Centro de estudios de Reproducción y Fertilidad humana
•	Instituto Tersoglio
•	Centro Integral de Ginecología, Obstetricia y Reproducción (CIGOR)
•	Centro de Investigaciones en Medicina Reproductiva (CIMER)
•	Centro de Estudios en Reproducción y Procedimientos de Fertilización Asistida (CRECER)
•	FERTILAB
•	GESTAR
•	Centro de Reproducción Fertilequip
•	Fertya
•	Gens, Centro especializado en tratamientos para la mujer
•	Hospital de Clínicas
•	FECUNDART
•	Centro de Reproducción, servicio de Ginecología Hospital Italiano
•	Mater, Medicina Reproductiva
•	Nascentis, Medicina Reproductiva
•	HALITUS, Instituto Médico
•	Instituto Medico de ginecología y Fertilidad PREFER
•	PREGNA, Medicina Reproductiva
•	Programa de asistencia reproductiva PROAR
•	PROCREARTE
•	Fertilidad San Isidro
•	SARESA, Salud reproductiva Salta
•	SEREMAS
•	VITAE, Medicina Reproductiva

BOLIVIA	
•	CENALFES
•	Instituto de Salud Reproductiva (ISARE)
•	EMBRIOVID, centro integral de reproducción y especialidades médicas

BRAZIL	
•	ANDROLAB, Clinica y Laboratorio de Reproducción Humana y Andrología
•	ANDROFERT, Centro de Referencia en Reproducción Masculina
•	FERTIVITRO, Centro de Reproducción Humana
•	BIOS, Centro de Medicina Reproductiva
•	FIV-MED
•	Centro de Medicina Reproductiva
•	VIDA, Centro de Fertilidad
•	Clinica FERTWAY
•	NASCER, medicina reproductiva ltda.
•	ORIGINARE, Centro de Investigación y Reproducción Humana
•	CLINIFERT, Centro de Reproducción Humana
•	CONCEPTUS, Centro de Reproducción Asistida de Cear
•	CONCEBER, Centro de Medicina Reproductiva
•	Clinica Origen
•	Clinica Pro-Genesis
•	Centro de reproducción humana CONCEPTION
•	Centro de Reproducción Humana MONTELEONE
•	Fértile Diagnósticos
•	CEERH, Centro especializado en Reproducción Humana
•	Embrios, centro de reproducción humana
•	EMBRYOLIFE, Instituto de Medicina Reproductiva
•	Centro de Reproducción Humana, Endoscopia y Medicina Fetal de Bahía (CENAFERT)
•	Instituto VERHUM
•	Clinica FERTIBABY BH
•	Fertilcare, Medicina reproductiva
•	FECUNDA, Reproducción Humana
•	FELICCITA, Instituto de Fertilidad Ltda.
•	HUMANA, Medicina Reproductiva (Ex centro de Reproducción asistida FEMINA)
•	FERTILITY, Centro de Fertilización Asistida de Campo Grande
•	FERTILITY, Centro de Fertilización Asistida
•	FERTIL Reproduccion Humana
•	REPROFERTY
•	FERTICLIN, Clínica de Fertilidad Humana
•	GENESIS, Centro de Asistencia en Reproducción Humana
•	Clinica Genics, medicina reproductiva y genómica
•	FERTIPRAXIS, Centro de Reproducción Humana (Ex Fert. Gin. y Obst. de Barra)
•	GERA, Grupo de endoscopia y Reproducción Asistida
•	Clinica GERAR VIDA
•	Instituto de Saude Da Mulher, Cegonha Medicina Reproductiva
•	IVI Sao Paulo, Chedid Grieco S.A.
•	HUMANA (PRIMORDIA, Medicina Reproductiva Huntington RJ)
•	Hospital de Clínicas de Riberao Preto
•	HUNTINGTON Campinas
•	HUNTINGTON, Centro de Medicina Reproductiva (Sao Paulo)
•	JULES WHITE, Centro de Medicina Reproductiva
•	HUNTINGTON Vila Mariana
•	Ideia Fertil
•	IMR, Instituto de Medicina Reproductiva e Fetal
•	Servicio de Reproducción Humana Del Hospital y Maternidad Santa Johana
•	Life reproducción humana
•	FERTILITAT, Centro de Medicina Reproductiva
•	Clínica MATRIX
•	Pro-criar Monte Sinaí
•	Centro de Reproducción Humana Nilo Frantz
•	Clínica ORIGEN
•	Procriar, Clinica de Fertilización Asistida, Blumenau
•	Clínica PRO-CRIAR, Medicina Reproductiva
•	Clínica PRO NASCER
•	Centro de Reproducción Humana De San Jose de Rio Preto
•	GENESIS, Centro de Reproducción Humana
•	Centro de Reproducción Humana Prof. Franco Junior
•	Centro de Ensino y Pesquisa en Reproducción Asistida (Centro de Rep. Asist. Hospital Da ASA SUL)

CHILE	
•	UMR Clínica de la Mujer Antofagasta
•	Centro de Estudios Reproductivos (CER)
•	Unidad de Medicina Reproductiva, Clínica Alemana
•	Unidad de Medicina Reproductiva, Clínica las Condes
•	Unidad de Medicina Reproductiva, Clínica de la Mujer
•	IVI Santiago de Chile
•	Programa e Fertilización Asistida I.D.I.M.I.
•	Clínica Monteblanco
•	Centro de Fertilidad y Medicina Reproductiva Concepción S.A.
•	Centro de reproducción humana, Valparaiso

COLOMBIA	
•	Centro FECUNDAR, Cali
•	Unidad de fertilidad del Coutry ltda. CONCEPTUM
•	Asociados en Fertilidad y Reproducción Humana
•	FERTIVIDA
•	Clinica Machicado SAS
•	Centro Médico IMBANACO
•	Instituto de Fertilidad Humana S.A.S. (INSER)
•	IN SER, Instituto Antioqueño de Reproducción
•	Procrear
•	Profamilia Fertil
•	Unidad de Fertilidad, Procreación Medicamente Asistida
•	Union temporal IN SER eje cafetero

ECUADOR	
•	Clínica de Medicina Reproductiva BIOGEPA
•	Centro Ecuatoriano de reproducción humana
•	Clínica INFES
•	Instituto Nacional de Investigación de Fertilidad y Esterilidad (INNAIFEST)
•	CONCEBIR, Unidad de Fertilidad y Esterilidad
•	Unidad de Fertilidad Hospital Alcívar

GUATEMALA	
•	Centro de Reproducción Humana S.A. (CER)

MEXICO	
•	Biofertility Center
•	Centro de Diagnóstico Ginecológico
•	URA, Unidad de reproducción asistida de Hispital CIMA Hermosillo
•	Instituto para el estudio de la Concepción Humana IECH
•	Centro de Reproducción Asistida del Hospital Español (HISPAREP)
•	Centro de Reproducción Asistida del Occidente
•	Centro de Reproducción Asistida de Saltillo
•	Centro Universitario de Medicina Reproductiva
•	CREASIS SC
•	Fertility Center Cancún
•	Ginecología y Reproducción Asistida GYRA
•	Grupo de reproducción y genética AGN y asociados
•	Instituto para el estudio de la concepción humana de Baja California
•	Instituto Mexicano de Alta Tecnología Reproductiva S.C. (INMATER)
•	Instituto de medicina reproductiva del Bajío IMER, sede Guadalajara
•	Instituto IMER de Tijuana
•	Instituto Mexicano de infertilidad
•	Instituto Médico de la mujer (RED CREA)
•	Instituto de Ciencias en Reproducción Humana, sede Guadalajara
•	Instituto de Ciencias en Reproducción Humana, sede Matamoros
•	Centro especializado para la atención de la mujer (CEPAM)
•	INGENES
•	INGENES Guadalajara
•	Unidad de reproducción humana y genética poliplaza médica
•	Instituto de Ciencias en Reproducción Humana (VIDA), sede León
•	Medica Fertil
•	Instituto de ciencias en reproducción humana del Sureste (Vida Merida)
•	Clinica Nascere
•	Centro de Medicina Reproductiva FILIUS
•	PROGEN, Reproducción asistida y medicina fetal
•	Clinica de Infertilidad y reproducción asistida de Toluca SA de CV
•	Centro especializado en esterilidad y Reproducción Humana (CEERH)
•	Instituto de Ciencias en reproducción humana VIDA, ciudad de Mexico.

NICARAGUA	
•	Centro de Fertilidad de Nicaragua

PANAMA	
•	IVI Panamá S.A.
•	Centro de reproducción Punta Pacífica
•	Instituto de salud femenina

PARAGUAY	
•	Neolife, Medicina y cirugía reproductiva

PERU	
•	Clínica CEFRA, Centro de Fertilidad y Reproducción Asistida
•	CERFEGIN
•	Centro de Fertilidad y Ginecología del Sur (CFGS)
•	Clinica de fertilidad del norte, Clinifer de Chiclayo
•	FERTILAB, Laboratorio de Reproducción asistida
•	Inmater, Clinica de fertilidad
•	Nacer
•	Grupo Pranor, Clínica CONCEBIR
•	Grupo Pranor, Instituto de Ginecología y Reproducción
•	Pranor, laboratorio de medicina reproductiva sede trujillo

REPUBLICA DOMINICANA	
•	Instituto de reproducción y ginecología del Cibao (IREGCI)

URUGUAY	
•	Centro de Esterilidad Montevideo (CEM)

VENEZUELA	
•	FERTILAB
•	UNIFERTES
•	Centro Medico docente la Trinidad
•	EMBRIOS, Centro de Fertilidad y Reproducción Humana, Hospital de Clínicas de Caracas
•	GENESIS, Unidad de Fertilidad y Reproducción
•	Instituto Venezolano de Fertilidad
•	Laboratorios In Vitro de Venezuela
